# The health for hearts united dissemination Trial: Implementation costs to reduce cardiovascular risk in African Americans

**DOI:** 10.1016/j.puhip.2025.100633

**Published:** 2025-06-26

**Authors:** Jon C. Mills, Jeffrey Harman, Pauline Muturi, Christina Davis, Iris Young-Clark, Penny Ralston

**Affiliations:** a Florida State University, College of Medicine, Department of Behavioral Sciences and Social Medicine, 1115 West Call Street, Tallahassee, FL, 32306, USA; b Center on Better Health and Life for Underserved Populations, Florida State University, P.O. Box 3061491, Tallahassee, FL, 32306-1491, USA; c Office of Career Development Services, School of Business and Industry, Florida Agriculture and Mechanical University, 1601 S. Martin Luther King Jr Blvd, Tallahassee, FL, 32307, USA

**Keywords:** Cost analysis, Cardiovascular disease, Church-based health, Community-based participatory research, Dissemination research, African Americans

## Abstract

**Objectives:**

We report on the implementation costs of disseminating Health for Hearts United (HH), a church-based intervention designed to reduce CVD in African Americans.

**Study design:**

Cost analysis from dissemination trial of the CVD risk reducing, HH Intervention.

**Methods:**

Total costs included materials purchased and labor hours contributed by the academic team to implement the intervention. Materials costs included supplies and printing calculated in total, as well as on a per-participant basis. Labor hours were tracked for each team member. Labor hours were further categorized by the phase of the intervention (Training, Planning & Coaching, Delivery & Recognition). Per-participant and per-church costs are reported as the cost measurement divided by the total health leaders that participated (reached).

**Results:**

A total of n = 168 out of 173 health leader participants were reached (97 %). Total program costs were $87,207.66. Total material costs were $13,308.00, while labor costs accounted for 85 % of the total program costs ($87,207.66) at $73,899.66. The Training Phase comprised the largest portion (74 %) of the total labor costs ($54,598.29). Total per-health leader participant reached cost were $519.09.

**Conclusions:**

In one of the first studies to report the costs of implementing a CVD risk reducing intervention among African Americans in a church setting, in partnership with a local academic institution, training was the main cost driver. Costs of implementing HH could be reduced by lowering hourly labor cost. Future research should examine costs associated with different methods of implementation (e.g., using more lay people).

## 1 Introduction

Cardiovascular disease (CVD) continues to be a major problem in the United States. African American adults experience higher morbidity and mortality rates for cardiovascular disease when compared to other ethnic/racial groups. For example, African American males and females have higher mortality rates in comparison to Non-Hispanic Whites [1]. Community-based programs in organizations such as churches have been recommended by Healthy People 2030 to reach African American populations [2]. Further, there is considerable literature that shows that church-based health programs are effective in improving cardiovascular-related health of African Americans [[3], [4], [5], [6]], yet few studies have examined the cost of implementing these programs within the context of an academic-community partnership.

To date the bulk of the cost-effectiveness research in relationship to interventions designed to reduce CVD-related morbidity has focused on studies implementing the Diabetes Prevention Program (DPP) [[6], [7], [8]]. In particular, Yeary et al., 2020 examined the costs of implementing an adapted version of the DPP in rural African American churches and found the intervention to cost $63 per pound lost by participants [6]. However, to our knowledge, this is the only study that has examined the cost of implementing an intervention designed to prevent obesity-related morbidity (diabetes) in an African American church setting. Moreover, there is also a paucity of research that examines these costs within context of academic-community partnerships that seek to sustain ongoing programming to improve health. Thus, the purpose of this paper is to report on the costs of disseminating a church-based intervention, Health for Hearts United [[9], [10], [11]] which was implemented through an academic-community based research partnership with the goal of reducing CVD risk in African Americans in North Florida.

## 2 Methods

### 2.1 Study Design

The Health for Hearts United (HHU) Dissemination Trial grew out of a larger academic-community partnership to reduce CVD risk through determining the effectiveness of a church-based intervention [[9], [10], [11]]. The HHU intervention, described in detail in Ralston et al. (2022), resulted from a planning session held with church health leaders following the completion of the effectiveness study. The model developed included three phases: a) Training, b) Coaching & Planning, and c) Delivery & Recognition.

• The Training phase included three sessions that were 2 h in length and included topics centered on the four key messages of the initial intervention developed in the Reducing CVD Risk Study: take charge of your health, eat better, move around more, and reducing stress [10]. The trainings also included brief presentations from health leaders who shared best practices on how they had implemented health programming related to the key messages.
• The Coaching & Planning phase included three components which were discussed in a 1-h planning meeting: individual health mentoring, health ministry planning, and CVD awareness event planning. Individual health mentoring included providing support for health leaders via telephone calls following the training phase to help them in moving forward in health behavior change with specific use of the monitoring tool, Health Check Report Card [12]. Health ministry planning included each health leaders team working on strategic plans to develop their health ministries. The final component of planning was a discussion with health leaders about their ideas for the CVD awareness event. The intent of planning and implementing was to build organizational capacity for health programming.
• The Delivery & Recognition phase focused on health leaders creating an event that would fit the church organization and using their influence to get support for implementation at the church level. This phase also included recognition of health leaders to build sustainability in health programming.

During the HHU Dissemination Trial, we recruited 173 health leader participants from 30 churches. The program was implemented with 7 cohorts, each comprised of a portion of the participants from participating churches.

Outcome Measure The outcome for assessing the impact of the program is the number of health leaders reached out of the number recruited to participant. This figure was calculated as the portion of health leaders that participated divided by the proportion of health leaders that were recruited to participate in the program.

### 2.2 Cost measures

We tracked costs for materials purchased and labor hours contributed by members of the academic team to intervention implementation efforts. Materials costs included supplies and printing costs calculated both in total, as well as on a per-participant basis. Labor hours were tracked for each project team member from the university, including the principal investigator, project coordinator and project assistants. Labor hours were further categorized by the phase of the intervention (Training, Planning & Coaching, Delivery & Recognition) in which the hours were accrued. For salaried project team members, we converted biweekly pay into an hourly rate by dividing the total biweekly amount by 80, assuming a 40-h work week. We used the given hourly rate of pay for project assistants paid by the hour. The total cost of labor was calculated by multiplying the hourly rate for each team members by the number of hours they reported contributing to each of the study's phases. Cost of labor is reported in total and by phase of intervention. Additionally, we calculated a total program cost by summing the total material costs with the total labor costs.

In addition to total costs, we report per-participant costs by dividing the cost figure by the total number of health leader participants reached (n = 168). Finally, a per church cost is calculated for labor hours by dividing the labor cost by the total number of participating churches (n = 30).

## 3 Results

### 3.1 Health leader participants reached - demographics

A total of 173 health leaders were recruited to participate in the HHU Dissemination Trial of which 70 % and 30 % were female and male respectively. The plurality (42 %) of health leaders were between the age of 43 and 63 years of age. The program reached 97 % of the health leaders, with 168 of those recruited participating in the program through follow-up (see Table 1).

### 3.2 Total program costs

The total program costs (See Table 2) of the HHU Dissemination Trial were $87,207.66, representing the sum of the total material ($13,308.00) and total labor costs ($73,899.66). Training was the largest cost component of the intervention, representing 63 % of total program costs. The total program cost per-participant reached as $519.09.

**Table 1:** Descriptive statistics of Health Leaders participating in The Health for Hearts United (HHU) Dissemination Trial.

Characteristic	Baseline	
	n = 173	%
Sex		
Female	121	70 %
Male	52	30 %
Age Category		
18–28 years old	26	15 %
29–42 years old	36	21 %
43–63 years old	72	42 %
64–77 years old	32	18 %
78 + years old	7	4 %
Total Reach	168	97 %

**Table 2:** Total program costs.

Cost Category	Amount	% of Total Program Cost	Cost Per Health Leader
Material Costs (Table 2)			
Printing	$5522.24	6 %	$32.84
Supplies	$7785.76	9 %	$46.37
Subtotal	$13,308.00	15 %	$79.21
Labor Costs (Table 3)			
Training	$54,598.29	63 %	$324.99
Coaching & Planning	$2600.01	3 %	$15.48
Delivery & Recognition	$16,701.36	19 %	$99.41
Subtotal	$73,899.66	85 %	$439.88
Total Program Costs	$87,207.66	100 %	$519.09

### 3.3 Material costs

Results for Material Costs are presented in Table 3. The total material costs (printing and supplies) for the HHU Dissemination Trial were $ 13,308.00, while the total material costs per-participant was $79.18. Supplies accounted for 59 % of the total material costs. Such costs included items to create participant binders, envelopes for surveys, items needed for the recognition event (e.g. church certificates, food). Printing comprised 41 % of the total material costs. Items included in the printing costs were materials for participants (e.g., notebook handouts, program booklet, planning handouts, consent forms, surveys), as well as certificates and church plaques.

**Table 3:** Material costs.

Printing	Unit Cost	Quantity	Total	Per-Participant	% of Total Material Cost
Notebook Handouts	$2.52	168	$423.36	$2.52	
Postcards^a^	$2.05	1176	$2410.80	$14.32	
Planning Handouts	$0.07	4	$47.04	$0.28	
Program Booklet	$1.05	168	$176.40	$1.05	
Pre and Post Surveys & Consent Forms	$1.40	168	$235.20	$1.40	
CVD Event Survey & Registration Sheets	$0.73	168	$122.64	$0.73	
Certificates	$1.56	168	$261.80	$1.56	
Church plaque	$61.50	30	$1845.00	$10.98	
Subtotal	–	–	$5522.24	$32.84	41 %

Supplies	Unit Cost	Quantity	Total Cost	Per-Participant	% of Total Material Cost

Participant Binder	$2.08	168	$349.44	$2.08	
Protective Sheets	$0.37	168	$62.16	$0.37	
Dividers	$1.25	168	$210.00	$1.25	
Survey Envelopes - Large Envelope	$0.44	168	$73.92	$0.44	
Survey Envelopes - Smaller Envelope	$0.63	168	$105.84	$0.63	
Framed Plaque	$44.99	30	$1349.70	$8.03	
Certificate Covers (Church)	$9.99	30	$299.70	$1.78	
Table Cover for HHU	$295.00	1	$295.00	$1.76	
Food	$30.00	168	$5040.00	$30.00	
Menu 1 - Traditional					
Menu 2 - Breakfast Burritos					
Menu 3 - Sausage & Cheese Casserole					
Subtotal			$7785.76	$46.34	59 %

Material Total			$13,308.00	$79.18	100 %

a Calculated at 2.05 post card ∗ 7 post cards per health leader.

### 3.4 Labor costs

Results for Labor Costs are presented in Table 4. Labor costs accounted for 85 % of the total program costs ($87,207.66) at $73,899.66. The Training phase comprised the largest portion (74 %) of the total labor costs ($54,598.29), with the Coaching & Planning phase comprising the smallest portion of total labor costs at $2600.01, representing 4 % of the total labor costs. Additionally, the Delivery & Recognition phase made up 23 % of total labor costs at $16,701.36. Finally, labor costs per-participant reached were $439.88 while the average per church cost for labor hours was 2463.32.

**Table 4:** Labor costs.

Phase	Type of Labor	Total	Cost Per Health Leader	% of Total Labor Costs
Training	Salaried	$45,468.29		
	Hourly	$9130.00		
	Sub - Total	$54,598.29	$324.99	74 %
Coaching & Planning	Salaried	$1949.01		
	Hourly	$651.00		
	Sub - Total	$2600.01	$15.48	4 %
Delivery & Recognition	Salaried	$13,165.36		
	Hourly	$3536.00		
	Sub - Total	$16,701.36	$99.41	23 %
Total Labor		$73,899.66	$439.88	

Total Labor Cost/Church (n = 30)		$2463.32		

## 4 Discussion

In this study, we report on the material and key study personnel labor costs associated with implementing the Health for Hearts United (HHU) Dissemination Trial across 30 churches with predominantly African American congregants in North Florida. To our knowledge this is one of the first studies to report costs of implementing an intervention to reduce CVD risk in the church setting, in partnership with the local academic institution. Total costs for implementing the HHU Dissemination intervention were $87,207.66. We found material costs accounted for 15 % of total program costs while labor comprised 85 % of total program costs. Training, a sub-category of labor costs was the largest driver of implementing the intervention, representing 63 % of total program costs. Finally, the total program costs of health leaders reached was $519.09.

The results of this study contribute to the limited literature on the cost of implementing church-based interventions. As stated earlier, K.H.K. Yeary's (2020) study of implementing an adapted version of the DPP to prevent obesity-related morbidity in rural African American churches resulted in $63 per pound lost [6]. Our results, which report per-participant and per-church level, are not comparable since we are unable to report per person costs and expenses in relation to a health benefit. Yet our study does provide a baseline of information to build upon in terms of categories of costs for community-based interventions implemented in churches. For example, a key outcome of this study is that labor costs comprised 85 % of the total costs. This is not surprising given that the HHU Dissemination Trial used the Community Based Participatory Research (CBPR) approach, consistent with the initial Reducing CVD Risk Study to determine the effectiveness of the HHU intervention. The literature on the cost of implementing CBPR is emerging [13,14], especially within a cost/benefit conceptual framework [15]. Having economic costs, as presented in this study, will contribute to this literature by providing quantitative data to the cost/benefit framework, especially in relation to academic-community partnerships.

Another interesting finding is that, within the HHU Dissemination Intervention itself, training costs were 63 % of the total program costs. Although this may seem high in comparison to the other intervention components, the HHU Dissemination Trial was conducted by cohorts, allowing us to save costs by bringing health leaders across churches together to participate in the trainings rather than implementing on a per church basis. Additionally, this approach saved time and we were able to implement all 30 churches within the time frame of the funded project.

The other intervention components were of lower cost in relation to total program costs, with Coaching & Planning comprising only 3 % and Delivery & Recognition encompassing 19 %. These lower percentages reflect the time considerations emphasized in the meeting with the health leaders from the six churches from the initial study. The Coaching & Planning Phase was based primarily on one meeting with health leaders and some individual work to help initiate any needed health behavior change in their own lives. The health leaders had to prepare for the meeting by providing a draft of the Health Ministry Development deliverable (i.e., a brief strategic plan for the health ministry to help ensure sustainability) [11]. The Delivery & Recognition costs were primarily focused on staff working shoulder-to shoulder, per CBPR, to implement their CVD awareness event but the bulk of that work was on the health leaders themselves with staff serving as advisors. The recognition activities (breakfast or lunch) incorporated more materials and staff time to plan and implement. These results, especially the work of the health leaders as volunteers, point toward capacity building in the health leaders themselves. Brush et al. (2020), in their scoping review to identify indicators of partnership success in CBPR, highlight partnership capacity building as one of the most powerful indicators identified in the literature examined [16]. Given the literature and the results of this study, a possible implication is the need to begin measuring volunteer time in church-based intervention cost studies to determine the extent of time involved in participation as well as the benefits perceived by partners from this participation.

One of the main limitations of this study is that per-church costs estimates may include variance related to the fact the program was implemented on a cohort basis, where each cohort consisted of health leaders from multiple churches. Cohorts with a larger number of churches will have lower per-church implementation costs than cohorts with fewer churches included. As such the per church cost estimates should be interpreted with caution. Although, this is the case, this study still provides valuable information about the total costs of implementing a church-based intervention and the proportion of total costs associated with various components of the intervention.

## 5 Conclusion

Much of the cost of implementing a church-based intervention in partnership with the academic community is associated with labor costs, as opposed to materials. Thus, the total cost of implementing similar interventions can be reduced if the hourly cost of labor is lowered, such as using lay people or volunteers to assist with implementation. Future research should examine costs associated with different methods of implementation (e.g., using lay people and tracking their volunteer time) in addition to relating costs to a health benefit.

## Statements of ethical approval

All study protocols were reviewed and approved by local institutional review boards.

## Sources of funding

10.13039/100006545National Institute on Minority Health and Health Disparities, Award Number MD002807.

## Declaration of competing interest

The authors declare that they have no known competing financial interests or personal relationships that could have appeared to influence the work reported in this paper.
